# Association Between Vascular Endothelial Growth Factor (VEGF) +936C/T Polymorphism (rs3025039) and Preeclampsia Among Myanmar Pregnant Women

**DOI:** 10.1155/2024/7608096

**Published:** 2024-06-06

**Authors:** Khin Ei Ei Saw, Thit Sar Aye Mg Thann

**Affiliations:** ^1^ Department of Anatomy University of Medicine, Taunggyi, Shan 06017, Myanmar; ^2^ Department of Anatomy University of Medicine 2, Yangon, Myanmar

**Keywords:** polymorphism, preeclampsia, vascular endothelial growth factor, VEGF

## Abstract

**Background:** The vascular endothelial growth factor (VEGF) polymorphism is associated with preeclampsia since its abnormal expression plays an important role in vasculogenesis in placenta formation. Thus, this study is aimed at analyzing the association between VEGF +936C/T polymorphism and the risk of preeclampsia.

**Methods:** To assess the causal relationship, a hospital-based cross-sectional analytical study was carried out among 204 Myanmar pregnant women during the period of January 2018–September 2020. For data collection, a pretested, structured questionnaire was used. Blood samples were collected after obtaining consent, and then we studied the extracted gene by using polymerase chain reaction–restriction fragment length polymorphism (PCR-RFLP). The Statistical Package for Social Sciences version 18.0 was used for data management and analysis.

**Results:** The genotype CT variant among preeclamptic women was more than that of non-preeclamptic women (26.5% vs. 18.6%), but not significant (*p* = 0.180). The risk of preeclampsia among women with CT genotypes was 1.57 times higher than that of women with CC genotypes (OR (95%CI) = 1.57 (0.81, 3.06), *p* = 0.180). The minor allele frequency of the T allele was 15.2% in preeclamptic women and 9.3% in normal pregnant women. The risk of preeclampsia among T allele carriers is 1.49 times (95%CI = 0.80, 2.77) more than that of C allele carriers (*p* = 0.211). Among the preeclamptic pregnant women, the frequency of the CT genotype was 26.3% in the severe preeclamptic group and 26.9% in the mild preeclamptic group, while the frequency of the T allele was 13.2% and 13.5%, respectively. The frequency of either CT genotype or T allele was more or less the same in both groups, and there was no association between VEGF C/T polymorphism and the severity of preeclampsia. After logistic regression analysis on VEGF genotype and clinical parameters such as age, maternal body mass index (BMI), and neonatal birth weight, the risk of preeclampsia was 2.1 times higher in pregnant women with CT genotype compared to CC genotype (adjusted OR, 2.1; 95% CI, 0.9–4.5, *p* value −0.057).

**Conclusion:** There was no significant association between VEGF +936C/T polymorphism (rs3025039) and preeclampsia among Myanmar pregnant women. However, the findings of this study highlighted that individuals carrying either the CT genotype or the T allele are at a heightened risk of developing preeclampsia. Furthermore, it suggests a potential impact of the gene on the occurrence of preeclampsia, yet the data lacks sufficient evidence to establish statistical significance.

## 1. Introduction

Preeclampsia is a pregnancy-related multisystem disorder occurring after 20 weeks of gestation. If preeclampsia is untreated, convulsions (eclampsia) may occur. In severe cases, complications such as hemolysis, elevated liver enzymes, and low platelet counts (HELLP) may arise, constituting the HELLP syndrome which occurs in up to 20% of severe preeclampsia cases [1–3].

Preeclampsia, affecting 5%–7% of pregnant women, imposes significant morbidity and mortality, contributing to over 70,000 maternal deaths and 500,000 fetal deaths worldwide annually [4]. Preeclampsia is a leading cause of maternal mortality in developing countries including Myanmar. In Myanmar, 19% of maternal deaths were due to pregnancy-induced hypertension in 2017 [5].

Moreover, preeclampsia does not end with the delivery of the placenta. Preeclampsia is a systemic disease with widespread endothelial damage and holds the potential to influence future cardiovascular diseases rather than being a self-limited event [6].

Since the single causative factor of preeclampsia remains unknown, extensive investigations have been undertaken to find out the etiology of preeclampsia and the theories regarding the causation of the disease are numerous and diverse. One of the main hypotheses is based on the failure of trophoblastic differentiation due to compromised vasculogenesis. It leads to placental ischemia and hypoxia, resulting in the release of specific soluble factors (i.e., endoglin, angiopoietin, and ephrin family protein) into the maternal circulation; consequently, this process induces systemic endothelial dysfunction [7].

Vascular endothelial growth factor (VEGF) is a key angiogenic factor since it serves as a regulator of vasculogenesis and vascular permeability. Its influence extends to critical early events in pregnancy such as trophoblast invasion and placentation [7]. Considering the important roles of VEGF in pregnancy, the potential significance of VEGF gene polymorphisms as genetic markers for preeclampsia susceptibility becomes apparent. Emerging evidence shows that VEGF gene polymorphisms are involved in the regulation of VEGF protein expression, thus increasing an individual's susceptibility to preeclampsia [8].

To date, no single theory has comprehensively elucidated the pathogenesis of preeclampsia. However, the literature suggests a robust association between VEGF gene polymorphism and the risk of preeclampsia since VEGF initiates vasculogenesis in the placenta in coordination with other angiogenic factors [9].

The VEGF gene is located in chromosome 6p21.3 and comprises a 14-kb coding region with eight exons and seven introns [10]. Many polymorphisms within the VEGF gene have been identified, and these variations can lead to changes in transcription factor recognition sites, influencing transcriptional activation and consequently affecting protein production levels [11]. Among these polymorphic sites, −460 T/C and −2578A/C in the promoter region, +405G/C in the 5′-untranslated region (5′ UTR), and +936C/T in the 3′-untranslated region (3′ UTR) are recognized for their capacity to influence the protein expression of VEGF [12]. Of these, one important VEGF variant gene had an error in the deoxyribonucleic acid (DNA) code located at DNA position +936 [13]. The normal VEGF gene contained a cytosine nucleotide (C) at this +936 location, but the variant had erroneously replaced cytosine with a thymine nucleotide (T) [14].

The +936 C/T exchange leads to the loss of a potential binding site for transcription factor AP-4 (activating enhancer binding protein 4), although the functionality of this binding site remains unclear. AP-4 plays a pivotal role in upregulating the expression of various viral and cellular genes by specifically binding to enhancer sites. VEGF polymorphisms may affect VEGF levels and, hence, its activity. Variant T in VEGF +936C/T decreases plasma VEGF level. Subjects carrying this mutation have significantly lower VEGF plasma levels than subjects carrying the VEGF+936 CC genotype [13].

Several case-control studies have investigated the VEGF polymorphism as a possible risk factor for preeclampsia. Previous research had demonstrated varied outcomes, with some studies indicating a connection while others reported no significant association between the VEGF +936C/T polymorphism (rs3025039) and the risk of preeclampsia in different populations. Findings from some of these investigations revealed an association between VEGF polymorphism and preeclampsia [15–21]. However, some studies described that there was no significant association between VEGF +936C/T polymorphism (rs3025039) and the risk of preeclampsia [17, 22-24].

The current study is aimed at detecting the association of VEGF +936C/T polymorphism (rs3025039) with preeclampsia risk in Myanmar pregnant women. The findings of the present study would highlight the significance of genetic variations in VEGF as potential risk factors for preeclampsia among pregnant women in Myanmar. These findings not only contributed to the understanding of preeclampsia's genetic aspects but also opened avenues for therapeutic interventions in managing and addressing preeclampsia in this population.

## 2. Materials and Method

This study was conducted among a total of 204 pregnant women (102 with preeclampsia and 102 control). They were recruited at the Department of Obstetrics and Gynecology, North Okkalapa General and Teaching Hospital, South Okkalapa Women and Children Hospital, and Thingangyun Sanpya General Hospital, after obtaining written informed consent. This study was carried out from January 2018 to September 2020 only after getting approval from the Academic Board of the Post Graduate Studies (anatomy) and the Ethics Review Committee, the University of Medicine 2, Yangon (Protocol no. 17/ERC-2, 3-2018).

Preeclamptic patients were defined as having systolic blood pressure of ≥ 140 mmHg and/or diastolic blood pressure of ≥ 90 mmHg on two occasions ≥ 6 h apart after 20 weeks of gestation, but before the onset of labor, plus proteinuria of ≥ 2+ (dipstick method) or ≥ 0.3 g/24 h urine specimen. Severe preeclampsia was defined as a higher blood pressure of ≥ 160 mmHg systolic or ≥ 110 mmHg diastolic on two occasions ≥ 6 h apart and a proteinuria level of ≥ 5 g/24 h or ≥ 3+ by dipstick testing on at least two separate occasions [22].

Patient recruitment criteria were (1) a pregnant woman of age between 18 and 50 years with clinically confirmed preeclampsia as the case and (2) a pregnant woman with no history of preeclampsia carrying a full-term pregnancy with a healthy baby as the control. Pregnant women with preexisting hypertension, pregestational diabetes, and end-stage renal and liver diseases as well as serious infectious diseases were excluded from the study.

Venous blood samples were collected from both preeclamptic and non-preeclamptic women, and then DNA was extracted by using a QIAamp DNA Mini Kit (Catalog no. 51306, Qiagen, Germany) and stored at −20°C in the deep freezer for genotyping. Extracted DNA was used as templates for the detection of VEGF +936 C/T gene mutations. According to Papazoglou et al. [23], forward primer 5′-AAG GAA GAG GAG ACT CTG CGC AGA GC-3′ and reverse primer 5′-TAA ATG TAT GTA TGT GGG TGG GTG TGT CTA CAG G-3′ were used for the polymerase chain reaction (PCR) cycling condition, and the thermal cycler was programmed to create a PCR program. It included 1 cycle of the initial incubation phase at 94°C for 5 min, a total of 35 cycles of denaturation at 94°C for 40 s, annealing with specific primers at 64°C for 1 min, elongation with *Taq* DNA polymerase at 72°C for 40 s, and 1 cycle of the final extension phase at 72°C for 5 min to make sure the DNA strands were fully extended and held at 4°C.

Then, the PCR products were digested with Hin1II restriction enzyme and incubated at 37°C overnight to analyze the different genotypes of VEGF +936C/T polymorphism (rs3025039). After digestion, the PCR–restriction fragment length polymorphism (PCR-RFLP) products were analyzed by 2% agarose gel electrophoresis with a 100 base pair (bp) ladder, and the results were recorded by evaluating under a UV transilluminator. A single fragment of 208 bp was identified as homozygous normal (CC); three fragments of 208, 122, and 86 bp were identified as heterozygous (CT); and two fragments of 122 and 86 bp were identified as homozygous mutant (TT) genotype.

Differences in the VEGF genotype and allele frequencies between the study and control groups were analyzed by chi-square test and OR (95% CI). *p* < 0.05 was considered statistically significant. Statistical analysis was performed using Statistical Package for Social Sciences version 18.0 for Windows software (SPSS Inc., Chicago, IL, USA).

## 3. Results

The genotype and allele frequencies of the VEGF gene at +936 position in a total of 204 pregnant women (102 preeclamptic and 102 non-preeclamptic women) were examined. The estimated risk was examined by the odds ratio. The clinical and demographic characteristics of the two study groups are shown in Table 1.

Genotypes were found to be in Hardy–Weinberg equilibrium in both the study and control groups. According to the Hardy–Weinberg equilibrium calculation, there was no significant deviation of VEGF +936C/T genotype distribution from the Hardy–Weinberg equilibrium (*p* > 0.05) in both preeclamptic and control groups. The present study found that the frequency of heterozygous CT is higher in women with preeclampsia as compared to non-preeclamptic women. The frequency of CT genotype in preeclamptic and non-preeclamptic women was 26.5% and 18.6%, respectively. The risk of getting preeclampsia among women with the CT genotype was 1.57 times higher than that of women with the CC genotype. The homozygous mutant TT genotype was not found at all in either preeclamptic or non-preeclamptic groups. The frequency of the T allele was found to be 13.2% in preeclamptic women and 9.3% in non-preeclamptic women, while the frequency of the C allele was found to be 86.8% and 90.7%, respectively. The frequency of the T allele among women with preeclampsia was also higher than that of non-preeclamptic women. The risk of getting preeclampsia among T allele carriers is 1.49 times more than that of C allele carriers (Table 2). However, either CT genotypes or T allele carriers were not associated with the severity of preeclampsia (Table 3).

The findings of the present study pointed out that there was no statistically significant difference in both genotype and allelic frequency of VEGF +936C/T polymorphism (rs3025039) among cases compared to the control. However, either CT genotype or T allele carriers are more likely to cause preeclampsia but are not statistically significant.

We then performed logistic regression analysis on VEGF genotype and clinical parameters such as age, maternal body mass index (BMI), and neonatal birth weight. The occurrence of preeclampsia was 2.1 times higher in pregnant women with the CT genotype compared to the CC genotype (adjusted OR, 2.1; 95% CI, 0.9–4.5). However, the CT genotype of the VEGF +936C/T polymorphism (rs3025039) did not achieve a statistically significant association with preeclampsia (*p* value −0.057) (Table 4). Thus, no association between VEGF +936C/T polymorphism (rs3025039) and preeclampsia was observed.

## 4. Discussion

Preeclampsia is a leading cause of maternal and fetal morbidity and mortality worldwide, as well as in Myanmar. It is a multisystem disorder and pregnancy-specific syndrome occurring after 20 weeks of gestation. Since the single causative factor of preeclampsia remains unknown, numerous investigations have been performed to find out the etiology of preeclampsia.

Exciting discoveries in the last decade have contributed to a better understanding of the molecular basis of this disease. Studying how genes play a role in preeclampsia helps us find women who might have a higher risk, and we can give them special care during pregnancy. By knowing the genetic component of preeclampsia, the identification of new pharmaceutical targets and additional therapies may be aided in the future.

Several lines of evidence support the hypothesis that VEGF plays an important role in the pathogenesis of preeclampsia. VEGF is a key component in the regulation of vascular remodeling and the survival of cytotrophoblasts in the placenta.

Moreover, VEGF +936C/T polymorphism (rs3025039) has been studied in relation to various gynecological and obstetric disorders including preeclampsia in various populations; however, its role in Myanmar women has not been studied yet. The present study is aimed at finding out the genotypes of VEGF +936C/T in preeclamptic women in comparison with non-preeclamptic women and detecting the association of VEGF +936C/T polymorphism (rs3025039) with preeclampsia risk in Myanmar pregnant women.

This study analyzed the effect of a single nucleotide polymorphism of the VEGF gene at +936 position among 102 preeclamptic and 102 non-preeclamptic women. According to the Hardy–Weinberg equilibrium calculation, there was no significant deviation of VEGF +936C/T genotype distribution from the Hardy–Weinberg equilibrium (*p* > 0.05) in both preeclamptic and control groups. The genotype distribution of the VEGF +936C/T polymorphism (rs3025039) conformed to the Hardy–Weinberg equilibrium in both groups. If a locus conformed to the Hardy–Weinberg equilibrium, the samples were representative of the population.

Various published studies have been performed to find out the association between VEGF polymorphisms and preeclampsia in various ethnicities. Comparisons between the present study and various authors are shown in Table 5.

According to Table 5, the findings of the present study suggested that the genotype distribution of the VEGF +936C/T polymorphism (rs3025039) was similar to the Egyptian, Pakistan, and Indian populations. However, in the Korean and Chinese populations, a higher frequency of the VEGF +936 TT genotype was seen in the present study.

In addition, Qi et al. and Tian et al. also reported the frequency distribution for the T allele in cases versus control as 38.3% versus 25.8% and 27.8% versus 22.7%, respectively [18, 19]. Both studies were done on Chinese populations. When comparing those results with the present results, the frequency distribution for the T allele in the Chinese population revealed almost twice that of the Myanmar population.

The finding of the present study was consistent with that of Fatima, Waryah, and Ahmed [25], who described that there was no significant association between VEGF +936C/T polymorphism (rs3025039) and the development of preeclampsia among the Pakistani population (*p* = 0.57). Also, Papazoglou et al. found no significant association between genotypes in women with preeclampsia relative to controls in the Greek population (*p* = 0.09) [23].

Another study done by Attia et al. among Egyptian women also found that there was no statistically significant difference in the frequency of homozygous mutant genotypes (TT) of the VEGF +936C/T gene among cases compared to controls [17]. Similarly, Cunha et al. also reported that there was no significant association between the VEGF +936C/T polymorphism (rs3025039) and preeclampsia among the Portuguese population (*p* = 0.4) [24].

In contradiction to the present finding, Arora et al. found that the VEGF +936CC (wild type) genotype was significantly higher in the controls, while the CT (heterozygous) genotype was higher in the patients (McNemar's test *p* < 0.0001) [21]. Shim et al., who studied Korean women, also described that the distribution of genotypes of the +936C/T polymorphism was significantly different between women with preeclampsia and the control group (*p* = 0.001) [20].

Qi et al. also studied VEGF +936C/T gene polymorphism in the Chinese population. They reported that the TT genotype of the VEGF +936C/T polymorphism (rs3025039) had an increased risk of preeclampsia. VEGF +936C/T polymorphism (rs3025039) contributed to the pathogenesis of preeclampsia among the Chinese population, and this single nucleotide polymorphism could be a susceptibility biomarker for preeclampsia: OR (95%CI) = 2.49 (1.32–4.68), *p* = 0.005 [18]. Moreover, Tian et al. also reported that VEGF +936C/T TT genotype frequencies were significantly higher than those with the CC genotype (*p* = 0.002, OR = 1.68 (1.17–2.24)). The VEGF +936C/T TT genotype was found to be associated with the risk of preeclampsia in Chinese pregnant women [19].

The variations noted across diverse studies might arise from disparities in study design, sample size, ethnic distinctions, the presence of modifier genes potentially concealing the impact of the studied gene (genetic epistasis), or population-specific environmental modifiers. Moreover, VEGF is also expressed by the fetus and in the placenta. It is imperative to take into account the fetal genotype, as the manifestation of the preeclamptic phenotype could result from the interplay of maternal and fetal genotype polymorphisms in conjunction with environmental factors. Another point to consider would be the role of fathers in the development of preeclampsia, especially whether he was born from a mother with preeclampsia. Nevertheless, the association of the VEGF +936C/T polymorphism (rs3025039) with preeclampsia might be influenced by various confounding factors, including obesity, which might be linked to VEGF. In the present study, the analysis of potential confounding factors was unfeasible, leading to discrepancies with the other research findings.

Similar to the present study, Papazoglou et al. and Cunha et al. found no significant association between allelic frequencies in women with preeclampsia relative to controls [23, 24]. But, in contrast with the results of the present study, Papazoglou et al. reported that a statistically significant difference was found for allelic frequencies of the +936C/T polymorphism between women with severe preeclampsia and controls: OR (95%CI) = 2.70 (1.09–6.63), *p* = 0.019 [23].

But the findings of the present study were in contrast with those of Shim et al. [20], Attia et al. [17], and Tian et al. [19]. Shim et al. described that carriage of the +936 T allele was significantly more frequent in preeclamptic patients than in control subjects: OR (95%CI) = 2.06 (1.38–3.08) [20]. Attia et al. performed a study among Egyptian female subjects. The study revealed that regarding the severity of preeclampsia, the frequency of cases carrying the homozygous mutant +936 T allele was statistically significantly higher among mild cases compared to severe cases (17.1% vs. 4.8%, OR = 3.39, *p* < 0.05) [17].

Tian et al. performed a study of VEGF SNPs +936C/T in a Chinese population. They found that VEGF +936C/T T allele frequencies were significantly higher in preeclamptic women than those with normal pregnancy: *p* = 0.003, OR = 1.32 (1.09–1.69). The VEGF +936 T allele was found to be associated with the risk of preeclampsia in Chinese pregnant women [19].

The determination of the genetic predisposition to preeclampsia poses a challenge due to the number of confounding factors. The divergent outcomes observed could likely be attributed to variations in age and parity. Usually, studies exploring preeclampsia in women employ age-matched controls to compare genotype frequencies.

The susceptibility to preeclampsia might be due to interactions between two or more maternal genes, and the linkage disequilibrium of the VEGF +936C/T polymorphism should also be tested together with other possible candidate genes. Since the present study concentrated on maternal genotypes alone, further research needs to be conducted examining both the maternal and fetal genotypes to find out possible maternal-fetal genotypic interactions. Moreover, paternal genetic contributions also need to be considered in further research since there was some evidence of changing susceptibility to preeclampsia if the partner had changed.

Since the frequency of primigravida was higher in control than preeclamptic cases, this could not rule out a possible future preeclamptic pregnancy among CT genotype carriers of control pregnant women. Age and parity-matched case-control studies should also be considered since environmental modifiers related to age could possibly mask the effect of the studied gene, and primigravida could not rule out a possible future preeclamptic pregnancy.

As a limitation of the present study, the samples were collected only from those hospitalized at North Okkalapa General and Teaching Hospital, South Okkalapa Women and Children Hospital, and Thingangyun Sanpya Hospital; the sampling population might not reflect the whole population of Myanmar. Considering the sampling procedure, there were some limitations to this study, such as the lack of opportunity for the selection of an age-matched control group.

As a strength of this study, it is the first to gather data on the VEGF +936C/T genotype percentage in the Myanmar population. This data can be used for future research on the connection between VEGF variation and pregnancy-related diseases. Nonetheless, understanding the role of genes involved in preeclampsia will enable us to identify women at high risk and thus target specialized antenatal care to this group. The findings of the present study would highlight the genetic variation of VEGF and would provide valuable information that contributes to our understanding of the VEGF gene for future research endeavors.

## 5. Conclusion

According to the findings, there is no significant association between VEGF +936C/T polymorphism and preeclampsia. However, the findings of the present study showed that the genotype CT variant among women with preeclampsia was more frequent than that of non-preeclamptic women. The risk of getting preeclampsia among women with the CT genotype was 1.57 times higher than that of women with the CC genotype. The distribution of the T allele among women with preeclampsia was also higher than that of non-preeclamptic women. The risk of getting preeclampsia among T allele carriers is 1.49 times more than that of C allele carriers. After clinical parameters such as age, maternal BMI, and neonatal birth weight are adjusted, the occurrence of preeclampsia was 2.1 times higher in pregnant women with the CT genotype compared to the CC genotype (adjusted OR, 2.1; 95% CI, 0.9–4.5, *p* value −0.057). Thus, either CT genotype or T allele carriers are more likely to occur the preeclampsia, and there is an effect of the gene on the occurrence of preeclampsia.

## Figures and Tables

**Table 1:** Clinical and demographic characteristics of two study groups.

**Variables**	**Preeclamptic women (** **n** **u** **m** **b** **e** **r** = 102**)**	**Non-preeclamptic women (** **n** **u** **m** **b** **e** **r** = 102**)**
Age (years)	30.9 ± 6.5 (range 19–47)	26.3 ± 6.1 (range 18–43)
Median (28 years)		
≤ 28	42 (41.2%)	72 (70.6%)
> 28	60 (58.8%)	30 (29.4%)
Diagnosis		
Mild preeclampsia	26 (25.5%)	
Severe preeclampsia	76 (74.5%)	
Gravidity		
Primigravida	43 (42.2%)	64 (62.7%)
Multigravida	59 (57.8%)	38 (37.3%)
Gestational weeks of delivery		
≤ 36 weeks	29 (28.4%)	4 (3.9%)
> 36 weeks	73 (71.6%)	98 (96.1%)
Family history of preeclampsia		
Positive	15 (14.7%)	2 (2.0%)
Negative	87 (85.3%)	100 (98.0%)
Systolic blood pressure (mmHg)	151.3 ± 14.1	113.7 ± 8.1
140–160	61 (59.8%)	
> 160	41 (40.2%)	
Diastolic blood pressure (mmHg)	95.2 ± 10.0	75.1 ± 8.1
90–110	83 (81.4%)	
> 110	19 (18.6%)	
Maternal BMI (kg/m^2^)	30.2 ± 5.5	26.6 ± 4.0
Neonatal birth weight (kg)	2.8 ± 0.6	3.1 ± 0.4

*Note:* Data are either mean ± SD or *N* (%).

Abbreviation: BMI, body mass index.

**Table 2:** Frequencies of VEGF +936C/T mutations in all preeclamptic cases compared to controls.

	**Case (** **n** = 102**)**	**Control (** **n** = 102**)**	**p**	**OR (95% CI)**	**Chi-square**	**HWE**
	**n** **(%)**	**n** **(%)**				
Genotype						
(CT)	27 (26.5)	19 (18.6)	0.180	1.57 (0.81, 3.06)	1.796	> 0.05
(CC)	75 (73.5)	83 (81.4)				
Allele						
(T)	27 (13.2)	19 (9.3)	0.211	1.49 (0.80, 2.77)		
(C)	177 (86.8)	185 (90.7)				

Abbreviations: HWE, Hardy–Weinberg equilibrium; OR, odds ratio; VEGF, vascular endothelial growth factor.

**Table 3:** Frequencies of VEGF +936C/T mutations in preeclamptic cases according to severity of the disease.

	**Severe PE (** **n** = 76**)**	**Mild PE (** **n** = 26**)**	**p**	**OR (95% CI)**
	**n** **(%)**	**n** **(%)**		
Genotype				
(CC)	56 (73.7)	19 (73.1)	0.952	1.03 (0.38, 2.82)
(CT)	20 (26.3)	7 (26.9)		
Allele				
(T)	20 (13.2)	7 (13.5)	0.956	0.97 (0.39, 2.46)
(C)	132 (86.8)	45 (86.5)		

Abbreviations: OR, odds ratio; PE, preeclampsia; VEGF, vascular endothelial growth factor.

**Table 4:** Logistic regression analysis for outcome variable using exposure variable (genotype) adjusting age, maternal body mass index (BMI), and neonatal birth weight.

**Genotype**	**Case (** **n** = 102**)**	**Control (** **n** = 102**)**	**p** **value**	**Odds ratio (95% CI)**	**Adjusted odds ratio (95% CI)** ∗
CT	27 (26.5%)	19 (18.6%)	0.057	1.57 (0.81, 3.06)	2.1 (0.9–4.5)
CC	75 (73.5%)	83 (81.4%)			

^*^Adjusted by logistic regression analysis with maternal age, BMI, and neonatal birth weight.

**Table 5:** Summarized table for various studies on the association between VEGF +936 C/T polymorphism and preeclampsia.

**Reference**	**Sample size**		**CC**		**CT**		**TT**		**C allele**		**T allele**		**Population**
	**Case**	**Control**	**Case**	**Control**	**Case**	**Control**	**Case**	**Control**	**Case**	**Control**	**Case**	**Control**	
Papazoglou et al. [23]	42	73	26 (61.9%)	56 (76.7%)	15 (35.7%)	16 (22%)	1 (2.4%)	1 (1.3%)	67 (79.7%)	128 (87.6%)	17 (20.2%)	18 (12.3%)	Greek
Shim et al. [20]	110	209	58 (52.7%)	150 (71.8%)	45 (40.9%)	55 (26.3%)	7 (6.4%)	4 (1.9%)	161 (73.2%)	355 (84.9%)	59 (26.8%)	63 (15.1%)	Korean
Cunha et al. [24]	52	28	34 (65.4%)	22 (78.6%)	16 (30.8%)	5 (17.6%)	2 (3.8%)	1 (3.6%)	84 (80.8%)	49 (87.4%)	20 (19.2%)	7 (12.4%)	Portuguese
Attia et al. [17]	100	100	71 (71%)	78 (78%)	29 (29%)	22 (22%)	0 (0%)	0 (0%)	171 (85.5%)	178 (89%)	29 (14.5%)	22 (11%)	Egyptian
Qi et al. [18]	128	128	70 (54.7%)	84 (65.6%)	18 (14.1%)	22 (17.2%)	40 (31.2%)	22 (17.2%)	158 (61.7%)	190 (74.2%)	98 (38.3%)	66 (25.8%)	Chinese
Tian et al. [19]	108	108	67 (62.1%)	72 (66.6%)	22 (20.6%)	23 (21.6%)	19 (17.4%)	13 (11.9%)	156 (72.2%)	167 (77.3%)	60 (27.8%)	49 (22.7%)	Chinese
Fatima, Waryah, and Ahmed [25]	80	80	65 (82.5%)	62 (77.5%)	15 (17.5%)	18 (22.5%)	0 (0%)	0 (0%)	145	142	15	18	Pakistani
Arora et al. [21]	50	50	40 (80%)	48 (96%)	10 (20%)	2 (4%)	0 (0%)	0 (0%)	90 (90%)	98 (8%)	10 (10%)	2 (2%)	Indian
Present study	102	102	75 (73.5%)	83 (81.4%)	27 (26.5%)	19 (18.6%)	0 (0%)	0 (0%)	177 (86.8%)	185 (90.7%)	27 (13.2%)	19 (9.3%)	Myanmar

## Data Availability

Data used to support the findings of this study are included within the article.

## Abbreviations

°C: Degree Celsius
3′ UTR: 3′-untranslated region
5′ UTR: 5′-untranslated region
A: Adenine nucleotide
BMI: Body mass index
bp: Base pair
BP: Blood pressure
C: Cytosine nucleotide
CI: Confidence interval
DNA: Deoxyribonucleic acid
g: Gram
G: Guanine nucleotide
HELLP: Hemolysis, elevated liver enzymes, and low platelet counts
kb: Kilo base
kg: Kilogram
LDL: Low-density lipoprotein
mmHg: Millimeter mercury
n: Number
OR: Odd ratio
PCR-RFLP: Polymerase chain reaction–restriction fragment length polymorphism
SD: Standard deviation
SPSS: Statistical Package for Social Software
T: Thymine nucleotide
UV: Ultraviolet
VEGF: Vascular endothelial growth factor.
